# Pedestrian Crossing Intention Forecasting at Unsignalized Intersections Using Naturalistic Trajectories

**DOI:** 10.3390/s23052773

**Published:** 2023-03-03

**Authors:** Esteban Moreno, Patrick Denny, Enda Ward, Jonathan Horgan, Ciaran Eising, Edward Jones, Martin Glavin, Ashkan Parsi, Darragh Mullins, Brian Deegan

**Affiliations:** 1 Connaught Automotive Research Group (CAR), University of Galway, H91 TK33 Galway, Ireland; 2 Department of Electronic and Computer Engineering, University of Limerick, V94 T9PX Limerick, Ireland; 3 Valeo, Tuam, H91 Galway, Ireland

**Keywords:** pedestrian, crossing, intention estimation, infrastructure, forecasting, behaviour

## Abstract

Interacting with other roads users is a challenge for an autonomous vehicle, particularly in urban areas. Existing vehicle systems behave in a reactive manner, warning the driver or applying the brakes when the pedestrian is already in front of the vehicle. The ability to anticipate a pedestrian’s crossing intention ahead of time will result in safer roads and smoother vehicle maneuvers. The problem of crossing intent forecasting at intersections is formulated in this paper as a classification task. A model that predicts pedestrian crossing behaviour at different locations around an urban intersection is proposed. The model not only provides a classification label (e.g., crossing, not-crossing), but a quantitative confidence level (i.e., probability). The training and evaluation are carried out using naturalistic trajectories provided by a publicly available dataset recorded from a drone. Results show that the model is able to predict crossing intention within a 3-s time window.

## 1. Introduction

According to the World Health Organization, approximately 1.25 million people die every year as a result of a road traffic accident, with pedestrians representing 22% of fatalities [1]. Autonomous vehicles have the potential to reduce traffic fatalities by eliminating accidents that occur due to human error. However, urban environments present significant challenges for autonomous vehicles. Non-signalized intersections present a particular challenge where the ego car must interact with other road users who may behave in complex and unpredictable ways.

Accurate prediction of pedestrian movements in an urban environment is an extremely challenging task. Although pedestrian detection and tracking algorithms have an extensive research history [2,3,4], detecting and tracking pedestrians is just the first step towards human-like reasoning [5,6,7] with the ability to answer questions such as: is the pedestrian going to intersect with the ego-vehicle’s path? Therefore, prediction of pedestrian behaviour is important for the safe introduction of autonomous vehicles into busy urban areas. Pedestrian behaviour has been studied extensively and related studies have been categorized based on the output (i.e., objective) produced by their algorithms [8]: trajectory prediction, crossing intention, behaviour classification (walking, starting, stopping, standing, walking-crossing, running-crossing) [9,10], movement direction [11] and goal prediction. The focus of this work is on forecasting pedestrian crossing intention.

Pedestrian intent is defined as the future action of crossing (*C*) or not crossing (*NC*) the road. Anticipating pedestrian intention might result in smoother and safer maneuvers, such as avoiding an unnecessary stop when a pedestrian at the roadside is not showing a crossing intention. Pedestrian intent prediction is especially challenging in the case of pedestrians because they can change their speed and heading unexpectedly. Current designs of connected and autonomous vehicles rely heavily on sensors fitted to the vehicle (i.e., on-board sensors) [5,6,7,10,12,13,14,15,16,17,18,19,20,21,22,23,24]. This is extensively reflected in the literature and will be discussed in the next section. Many dangerous situations arise from the fact that the driver’s view of a pedestrian is restricted [24]. To help compensate for a the limited field of view from on-board sensors, fixed infrastructure sensors may be used. Furthermore, research initiatives are concerned with short-term and long-term predictions [8]. Short-term approaches predict pedestrians’ position between 2.5 and 3 s [25,26], while long-term predictions are more goal-oriented [8].

This study proposes a machine learning approach to predict pedestrian crossing intention up to 3 s before the road crossing takes place. A publicly available dataset recorded from a fixed drone is used to evaluate the model on naturalistic trajectories collected at an intersection. To our knowledge this is the first time pedestrian intention is estimated and evaluated from a drone perspective instead of on-board or ground-level infrastructure sensors.

The main contributions of this work are:

- Utilization of random forest (RF) for detailed pedestrian intention forecasting using naturalistic trajectories that were captured using a drone;
- An algorithm to automate the addition of pedestrian crossing intent labels to the existing dataset (see Section 4.1);
- Extensive and detailed validation and evaluation of pedestrian trajectories extracted from real data to show that the proposed model is applicable at different crossing locations, not just a predefined ROI (e.g., crosswalk). Scenarios where the crossing intention is not clear are also investigated (e.g., pedestrian slowing down before crossing);
- Comparison analysis between an RF and a feed-forward neural network (NN) in the context of pedestrian intention forecasting, verifying that the proposed approach outperforms the NN.

This paper is structured as follows. Section 2 reviews the related literature about pedestrian intent forecasting using data collected from on-board sensors or a fixed node. Section 3 describes the problem formulation and introduces the chosen dataset. Section 4 discusses how the data were pre-processed to add new labels that were used during training. It introduces a case study evaluating different naturalistic pedestrian trajectories (i.e., road users were not aware of measurements taking place) in the given intersection. Also, a comparison analysis between an RF and a feed-forward neural network (NN) is conducted. Section 5 concludes the findings and discusses future work.

## 2. Related Work

A mature pedestrian crossing intention system should consist of detection, tracking and intention recognition components [27]. A considerable body of literature exists on pedestrian detection and tracking [2,3,4,28,29,30,31,32,33] and while there is still scope for improvement, pedestrian detection algorithms are well developed thanks to convolutional neural networks (CNN) and common architectures such as YOLO [28] and Faster R-CNN [29]. Tracking algorithms using deep learning [30,31] are starting to reach the performance levels of traditional and state-of-the-art tracking methods [32,33]. Thus, in this section pedestrian crossing intention prediction is examined exclusively.

There is a clear distinction in the literature between predictions made with data collected from an on-board sensor and data collected from a fixed sensor in the infrastructure [34]. Consequently, the following section is partitioned according to this distinction.

### 2.1. On-Board Sensors

State-of-the-art automated driving systems employ a range of on-board sensors such as cameras, lidar and radar, although the majority of studies on pedestrian intent are based on video sensors normally placed behind the windshield of the vehicle [5,6,7,10,12,13,14,15,16,17,18,19,20,21,22,23,24]. 

A vision-based pedestrian intention prediction approach from monocular images is proposed in [7]. Using a multi-stage CNN [35], pedestrian pose is analysed for several frames to determine if they are likely to cross the road. The authors report a classification accuracy of 0.88. The evaluation is performed on a publicly available naturalistic dataset focused on driver and pedestrian behaviour. Intention estimation from a monocular dashboard camera is also addressed in [18]. A large-scale dataset designed for pedestrian intention estimation is proposed together with a baseline model for intention and trajectory estimation. The ground truth for crossing intention is collected by conducting a large-scale experiment to determine human reference data for this task. Their intention estimation model, based on a convolutional long short-term memory neural network (LSTM), achieves an accuracy of 0.79. In [6] graph convolution is used to model the spatiotemporal relationship between pedestrians and other objects of interest in the scene. A new dataset, designed specifically for autonomous-driving scenarios in areas with dense pedestrian populations, is also introduced. Results show that their spatiotemporal relationship reasoning model can predict pedestrian intention with an accuracy of over 0.8 on their own dataset one second before the crossing commences.

Contextual knowledge using derived features or prior knowledge of the environment [19,20] can improve prediction. A context-based model is introduced in [21] predicting pedestrian crossing behaviours in inner-city locations using a set of twelve carefully chosen features. A supervised learning algorithm is deployed and trained on automatically labelled the pedestrian’s crossing intentions. Context is also taken into consideration in [22], modelling pedestrian movement in relation to relevant road edges. In order to determine the relevant road edges, the scene is segmented into four zones comprising: the ego-zone (defined by the ego-vehicle lane), the street-zone (comprising all non-ego-vehicle lanes), the mixed-zone (parking lanes and inlets for buses) and the sidewalk-zone. The reported accuracy using a Support Vector Machine (SVM) is 0.897. Crossing behaviour at signalized intersections is modelled in [23] by incorporating pedestrian physical states and contextual information. The probabilistic model is constructed using a dynamic Bayesian network and is trained with data collected using three monocular cameras mounted on the car. A novel dataset is introduced in [12] to facilitate traffic scene analysis and pedestrian behaviour. The dataset is captured from three cameras positioned inside a car (below the rear-view mirror), providing contextual and behavioural information along with bounding boxes. In order to embed contextual information in the data, each frame is assigned a contextual tag describing the scene (e.g., number of lanes, presence of a zebra crossing, time of day or weather). Results show that classification using only the attention/gait information (i.e., behavioural information) can correctly predict approximately 40% of crossing behaviour observed; however, adding context, such as the width of the street and the presence of the designated crossing, improves the classification by 20%. Two-dimensional human pose features and scene context are investigated in [13] to forecast the crossing intention of pedestrians and other vulnerable road users (i.e., non-motorised road users). A multi-task learning model is proposed to predict actions, crossing intent and trajectories. Finally, pedestrian intent recognition, exploiting various sources of contextual information, is tackled in [14] as a time series modelling and binary classification problem. A novel stacked recurrent neural network (RNN) is proposed, achieving accuracies of 0.844. Moreover, a framework for pedestrian intention estimation is proposed and deployed on a real vehicle in [15]. Results from the field tests showed 0.75 overall accuracy. Additionally, using a Bayesian network the motion and intention of pedestrians near a zebra crossing is estimated in [16] with 91.8% and 84.6% success in motion and intention prediction, respectively, one second into the future.

In more recent work, [17] pedestrian crossing intention is determined though 3D Pose estimation, achieving 0.9128 accuracy. This sets a new standard of performance, although the *C*/*NC* labelling is done manually frame by frame. It should also be noted that the test and validation sets have only 7 pedestrians each.

### 2.2. Infrastructure Sensors

To date, not as much work has been done on crossing intention forecasting from infrastructure sensors (e.g., roadside sensors or cameras installed on aerial vehicles, such as drones) as there has been with on-board (on vehicle) sensors. Previous studies have focused on the detection and tracking of pedestrians and vehicles [36], pedestrian counting using video surveillance cameras [37], interactions between pedestrians and other agents or path prediction. In [38] interactions between pedestrians and vehicles are studied using video data from two cameras fixed to a lamppost. Their framework, also referred to as the distance-velocity model, is based on vehicle trajectory speed and distance to the crosswalk. In path prediction (i.e., trajectory forecasting), the forecasting of destinations is studied. Although strictly speaking this is not direct pedestrian intention estimation (i.e., binary intention interpretation (*C*, *NC*)) it is still classified as pedestrian behaviour prediction. In [26] trajectory prediction is also studied using real-world data collected from a static wide-angle camera and an artificial neural network (ANN). The ANN has two hidden layers and can forecast pedestrian short-time trajectories up to 2.5 s ahead of time for traffic safety applications. Results show that the ANN outperforms other methods such as a constant velocity model Kalman filter (KF) and the extrapolation of fitted polynomials by 21% on average. Using outdoor video recordings and pre-generated cost maps dependent on the environment semantics, an extension to the growing hidden Markov model is proposed in [19] to predict pedestrian positions over a longer time horizon.

Recently, there has been a trend towards formulating the problem of trajectory forecasting as a time series prediction problem using an RNN and its variants [39,40]. In [41] a model where a LSTM is used to predict human movement and forecast future trajectories is proposed. The LSTM method is demonstrated on two publicly available human-trajectory datasets and has been shown to predict motion dynamics in crowded scenes, outperforming methods such as an off-the-shelf KF, or social force model [42]. Based on the same datasets, [43] shows that a simple constant velocity model outperforms some of the most advanced neural network architectures when dealing with linear trajectories.

It is worth mentioning the work by [44,45]. In [44] the authors use quantile regression forests to predict time-to-cross when approaching a crosswalk. Their evaluation is based on vehicle and pedestrian tracks extracted from LIDAR data from a crosswalk in Germany. Because of their aim, prediction of pedestrian crossing behaviour at urban crosswalks in terms of remaining time-to-cross, they only evaluate the trajectories of actual crossing pedestrians. In [45] nine types of pedestrian-vehicle interactions are interpreted by using pose estimation to generate 2 d key points on a pedestrian skeleton and RF. The highest reported accuracy is 0.965 on their own dataset.

Nine studies on crossing intent prediction from a fixed sensor were identified in the literature. Using a database of LIDAR-derived pedestrian trajectories, a large set of features is studied by [46]. The best features are selected to train a binary classifier based on a Support Vector Machine (SVM) achieving an accuracy of 0.9167. In [47], the same authors propose a pedestrian motion prediction method combining pedestrian motion tracking algorithm and a data-driven method, which improves the generalization ability of the model. The pedestrian intention classifier is based on a SVM and mainly serves as a data reduction procedure removing irrelevant pedestrians from the scene, therefore reducing the number of targets to be tracked. For all identified crossing pedestrians, the time-to-cross and crossing point is predicted. Pedestrian intention to cross the road is also investigated by [24] to develop an active pedestrian protection system. Using motion contour histograms of oriented gradients combined with a SVM they estimated the pedestrian crossing intent. Using high-level pre-processed data (e.g., position, velocity, orientation), several ML algorithms are used in [48] to train different classifiers to estimate the intention of a pedestrian to cross at a zebra crossing. Four features are derived from the original dataset and used in the classification task: (i) distance to zebra crossing, (ii) distance between the nearest road border point and the pedestrian position, (iii) distance between the nearest road point and the zebra anchor, measured along the border curve and the angle between a pedestrian’s current heading direction, and (iv) pedestrian-to-zebra direction. The k-nearest neighbours is found to be the best performing method for classification, achieving a F2 score (i.e., test accuracy) of 0.9237. Using pre-processed roadside LIDAR point cloud data, a naïve Bayes based model is presented in [25], predicting the intention to cross between 0.5 and 3 s before the crossing commences. Only 3 features are selected: position, velocity and direction. For evaluation, a test set is collected and divided into 10 *C* and 10 *NC* trajectories using a probability threshold of 40%. Results show a high prediction accuracy and higher flexibility than the popular ANN model. LIDAR-based images (i.e., sampled from a point cloud), and different deep learning architectures are used in [49] to estimate pedestrian intention at a crosswalk. A precise map of the environment is utilized, providing information on road geometry and the accurate position of curbs and crosswalks. The best results are achieved using a dense neural network achieving an accuracy greater than or equal to 0.8. The use of LSTM or CNN architectures does not yield the expected improvement although all of them outperform the SVM, which is used as a baseline for performance evaluation. An LSTM is deployed in [50] to predict pedestrians red-light crossing intentions on video data collected at a crosswalk, achieving an accuracy of 0.916. Crossing intention is labelled manually along with other features such as gender, grouping behaviour and walking direction. The model presents a high false-positive rate and according to the authors this problem can be overcome by adding more features related to pedestrian mobility information such as walking speed and acceleration. An extension of the previous work is found in [51] where four machine learning models are used to predict pedestrians’ red-light crossing intentions. The best model, an RF, achieves an accuracy of 0.920. An LSTM with attention mechanism (AT-LSTM) is proposed in [27]. Attention mechanism (AT) ensures that greater weight is given to key features. Thanks to the addition of AT and precisely chosen parameter sets using statistical analysis, the model achieves accuracies of 0.9615 and 0.9068, 0 s and 0.6 s prior to crossing, respectively.

In this paper, we propose a model based on an RF that predicts pedestrian crossing intention at different locations around an urban intersection (i.e., crosswalk, pedestrian walking in parallel to the road and a pedestrian walking on other arms of the intersection), not just a predefined ROI. Random forests is a well-established algorithm for statistical learning and has been applied to classification and recently for classification of pedestrian trajectories [48,52,53] and intention [5,51]. We also investigate the addition of a derived categorical feature, which we have called *crossing*, to the original dataset by performing a semantic segmentation of the environment (i.e., partition the image into two segments: sidewalk and road), and evaluate ten crossing and ten non-crossing trajectories. Results show how effective the model is at predicting pedestrian crossing intention when the TTC is above 2 s. Furthermore, we conduct a comparison between an RF and a feed-forward neural network (NN), showing that the former outperforms the latter in terms of accuracy and training times. Finally, we study how the model reacts when the pedestrian demonstrates apparent hesitation (e.g., a significant velocity drop before crossing) and the potential impact of oncoming traffic.

## 3. Methodology

The problem of crossing intention prediction is formulated as a binary classification task where the objective is to determine if a given pedestrian will cross or not cross the street. The proposed model is able to predict the crossing intention of pedestrians walking at different locations around an unsignalized intersection (e.g., crosswalk, sidewalk or in front of the curb) given a list of selected features and derived features provided by a publicly available dataset and data preprocessing, respectively.

### 3.1. Random Forest

A random forest [54] is a supervised machine learning algorithm used for classification and regression. As an ensemble learning method, many decision trees are constructed during training using bagging [55] and feature randomness. Each decision tree, consisting of split and leaf nodes, is fitted with random sub-samples of the dataset. Once the RF is trained, the dependent variable is computed by averaging the prediction of all trees in the ensemble (i.e., forest), in the case of regression, or by majority vote of the predicted class label, for classification. This paper focuses on the latter, the classification task. Random forests can also deal with missing and categorical data [56], are less computationally expensive, are more interpretable and are less prone to overfit than a (NN) [57,58]. Neural networks trained models are difficult to interpret, consisting of hundreds to millions of parameters. This black box problem makes it complicated to use NN in applications where trustworthiness and reliability of the predictions are of great importance. Humans have to understand what models are doing, especially when they are responsible for the consequences of their application [59].

Given a set of input features *v*, a trained RF classifier will output the class c they belong to and the probability of it being in that class. The predicted class probabilities of an input feature vector are computed as the mean predicted class probabilities of the trees in the forest, denotated by T. The class probability of a single tree is the fraction of samples of the same class in a leaf [60].

(1) $$p\left(c|v\right)=\frac{1}{T}\overset{T}{\underset{t}{\sum }}p_{t}\left(c|v\right)$$

For the case of an RF, important hyperparameters include: number of decision trees in the forest and number of features considered by each tree when splitting a node. For the experiments, 500 tree estimators with $\sqrt{v}$ features in each split are used. To measure the quality of the split, entropy is used.

(2) $$Entropy=\overset{\#Classes}{\underset{i=1}{\sum }}-p_{i}*\;\log_{2}\left(p_{i}\right)$$

### 3.2. Dataset

**Figure 1:**
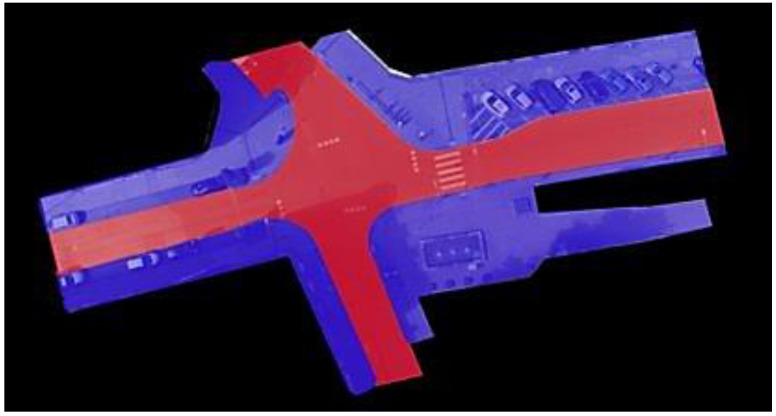
Segmented intersection. Blue represents the sidewalk and red the road.

This study relies on a publicly available dataset [61] to train and test the proposed model. Pedestrian trajectories are extracted from drone video recordings taken at four German road intersections. The videos were recorded in 4K (4096 × 2160 pixel) resolution at 25 frames per second. Every recording has a duration of approximately 20 min covering areas of 80 × 40 m to 140 × 70 m. All four intersections are unsignalized and differ in shape (e.g., T-junction, four-armed), right-of-way rules, and the number and types of lanes and traffic composition. In this study, the intersection at *Frankenburg* is chosen (see Figure 1) because it contains the largest number of pedestrians, due to its location near the city center. There are 12 recordings (#18–#29) of this intersection with a total duration of ~242.5 min. An image and three CSV files are provided per recording. The image shows the intersection from a drone’s viewpoint. Two of the CSV files contain meta data about the road users and the recording itself (e.g., recording number, track id, class, frame rate, duration, location, number of pedestrians) while the third contains the trajectory information. The features provided per frame are: *id*, *frame*, *xPosition*, *yPosition*, *xVelocity*, *yVelocity*, *Heading*, *xAcceleration* and *yAcceleration*. The dataset contains trajectories of vehicles including cars, buses and trucks, as well as VRU such as pedestrians and cyclists; however, in this work pedestrian trajectories are the primary interest.

Pedestrian crossing intention is inferred based on their features (*xPosition*, *yPosition*, *xVelocity*, *yVelocity*, *Heading*) and a derived feature *distanceToRoad*. As discussed in Section 2, derived features and semantic segmentation of the environment can improve prediction. Here, the derived feature *distanceToRoad* is extracted along with *x-yPosition*, *x-yVelocity* and *Heading*, to train an RF. Given a trajectory sample, the RF will output a class label *(C*, *NC)* and its probability. The next section discusses how the data are processed to extract the labels (*C* and *NC*) and presents results in two cases: when the model is applied to the overall dataset and when the model is applied to a particular subset of selected trajectories, belonging to one of the recordings of the chosen intersection.

## 4. Experiments and Results

### 4.1. Data Preprocessing

Since the original dataset does not provide labels for pedestrian crossing intention, an automatic labelling strategy (i.e., an algorithm that provides the ground truth at each timestep) was adopted. A similar approach was carried out by [21], although their data were collected from an on-board camera and they presented their results from the ego lane perspective.

**Figure 2:**
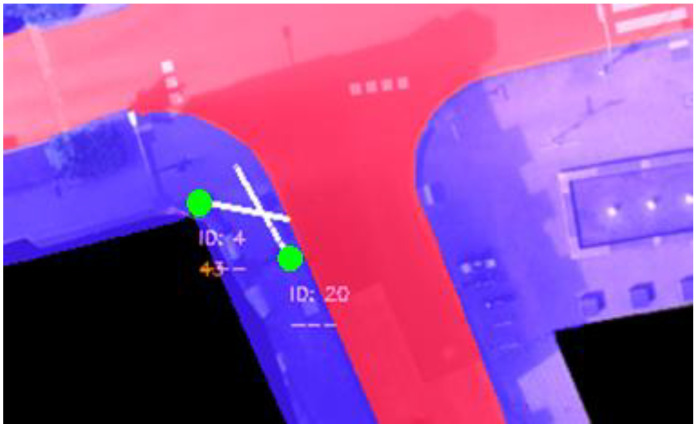
Distance to the road from current pedestrian position. The pedestrian current position is represented by the point shown in green and the heading direction by the white line.

In this paper, in order to add the derived categorical feature *crossing* to the original dataset, a basic semantic segmentation of the environment is needed to partition the image into two segments: sidewalk and road. This segmentation was done manually since the dataset did not provide a high-definition map, although it could have been done automatically using data from OpenStreetMaps [62] and a CNN [63] or using roadside LIDAR data [64]. Figure 1 shows the segmentation applied to the chosen intersection. As a result of this segmentation and a derived feature, which we have called *distanceToRoad*, it can be determined if a pedestrian commences crossing the road. The *distanceToRoad* feature can be calculated by casting a vector relative to the pedestrian’s *heading* from its current position to the road and measuring the vector length (Figure 2). When a crossing action is detected, each previous timestep (up to 3 s) is annotated as a *C* behaviour. The rest are considered *NC* except for the trajectories where the pedestrian is on the road (i.e., being on the road is always considered *C*) which are ignored and not used during training or testing.

**Figure 3:**
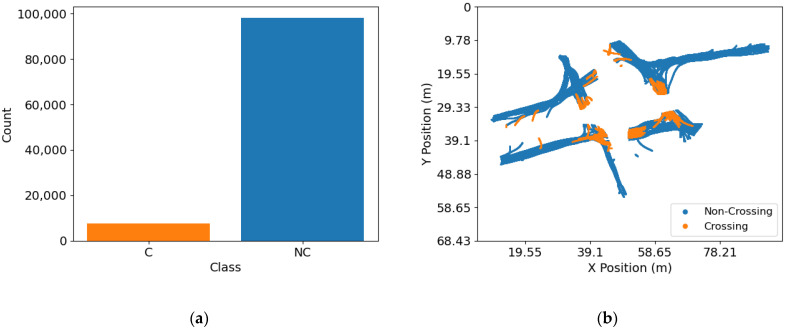
Dataset containing the derived categorical feature *crossing* for recording #18. (**a**) Imbalance among classes; (**b**) color-coded trajectories of *C* and *NC* trajectory samples.

In the dataset containing the derived categorical feature *crossing*, there is a significant imbalance among classes (*C*, *NC*). This imbalance can be observed in Figure 3 for recording #18, where ~7700 *C* samples and ~98,990 *NC* samples. The imbalance was addressed by randomly down sampling the majority class [65], *NC* in this case.

### 4.2. Training

Using crossing labels and pedestrian features *xPosition*, *yPosition*, *xVelocity*, *yVelocity*, *Heading* and the derived feature *distanceToRoad*, an RF was trained with the hyperparameters discussed in Section 3. For training and evaluation Scikit-learn was used [66] which is a Python machine learning library. The trajectory data were shuffled and split into training (70%) and test sets (30%) for model fitting and evaluation purposes. We also ensured the test set did not contain any duplicates of trajectory samples found in the training set. Eleven recordings from the intersection located at Frankenburg (#19–#29) were used. The pedestrian trajectory data from the recording that was not used for training (#18) was used to perform further evaluation.

### 4.3. Evaluation

**Figure 4:**
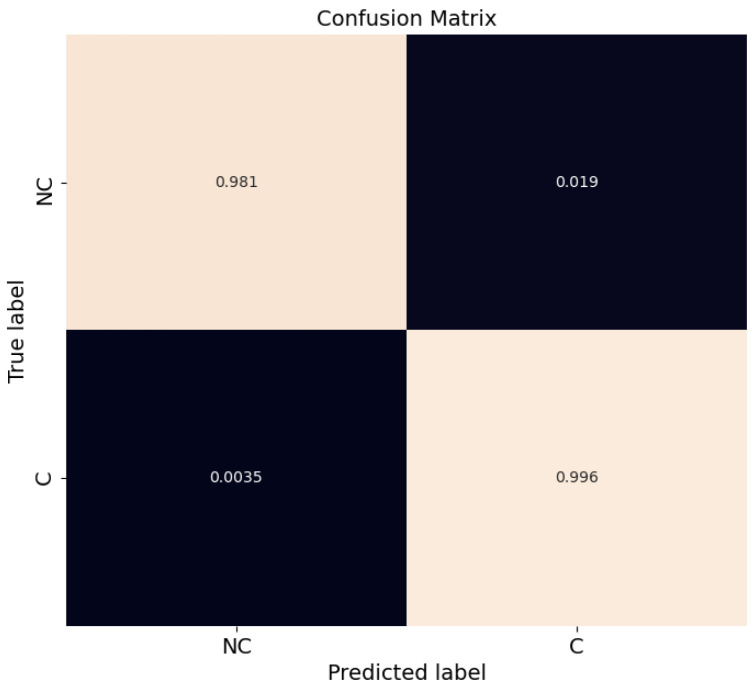
Confusion matrix for RF classification for test set (recordings #19 to #29).

Since a binary classifier was used, performance can be expressed by using a confusion matrix. This gives the number of correctly and incorrectly classified instances: true positives (TP), true negatives (TN), false positives (FP) and false negatives (FN). The normalized confusion matrix for the test set is illustrated in Figure 4.

Classification accuracy is also computed according to the widely accepted definition:

(3) $$Accuracy=\frac{\left(TP+TN\right)}{\left(TP+FP+FN+TN\right)}\;$$

*K*-fold cross-validation with *k* = 5 is used, which results in an average accuracy of 0.9883 with a standard deviation of 0.0006.

The confusion matrix in Figure 4 shows that the model is able to classify *C* and *NC* trajectory samples successfully, with TPs and TNs above 97%. In terms of FNs, which is an important safety concern, the number is below 1%. The FPs are also low at ~2%.

**Figure 5:**
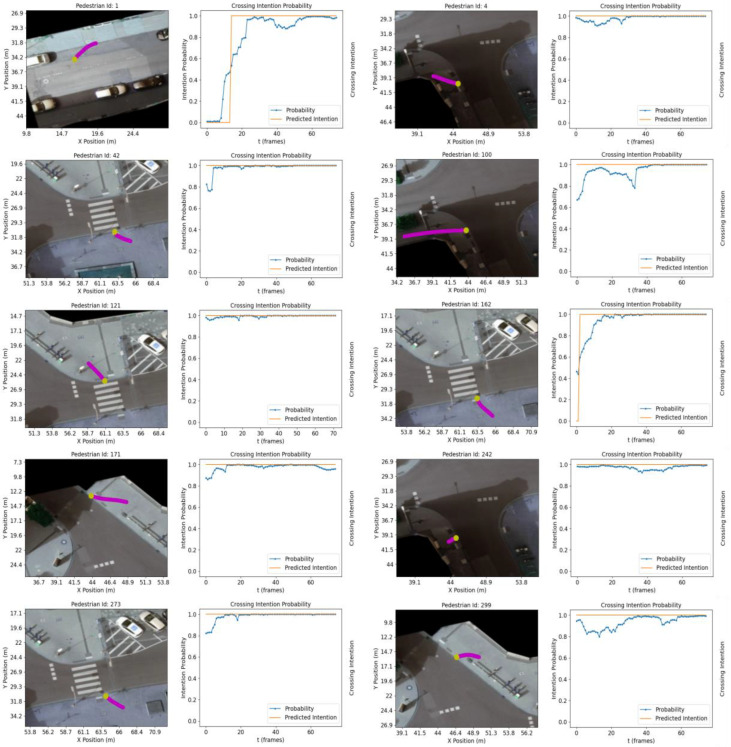
Classification results for 10 crossing trajectories. Pedestrian trajectories are shown in magenta. The trajectory point shown in yellow indicates the last trajectory sample in the sequence.

**Figure 6:**
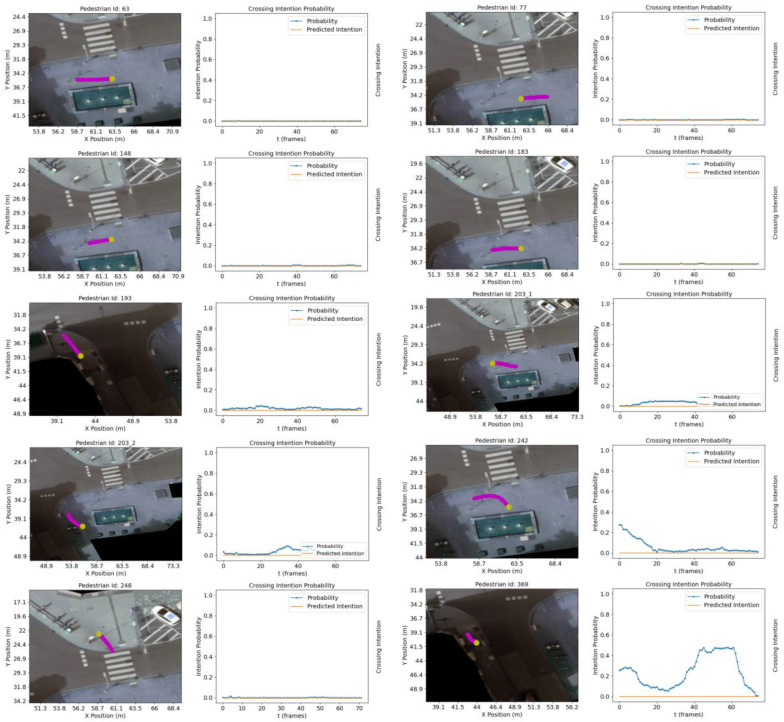
Classification results for 10 not crossing trajectories. Pedestrian trajectories are shown in magenta. The trajectory point shown in yellow indicates the last trajectory sample in the sequence.

To further evaluate the model performance, ten crossing and ten non-crossing trajectories, previously unseen by the model during training, are chosen in an experiment similar to that performed in [25]. The trajectories are chosen from recording #18. Figure 5 and Figure 6 show a subset of the selected *C* and *NC* trajectories along with the crossing intent prediction and its probability. The possible predicted intention values are 0 or 1, *C* and *NC*, respectively.

Our results show a higher confidence level than [25] when the TTC is longer than 2 s. As expected, the crossing probability increases as the pedestrian approaches the crossing facilities (e.g., zebra crossing or road edge). Prediction accuracy for the ten crossing trajectories is 0.97, with a decision boundary of 0.5. As in [25], intent prediction is computed every 0.5 s. In the evaluation for the ten non-crossing trajectories, it is not possible to replicate the experiment meticulously due to differences in the road structure (e.g., size of the sidewalk when approaching decision making point). Instead, NC trajectories capturing a range of scenarios are presented (e.g., pedestrians turning when approaching any of the crossing areas, pedestrians walking in parallel to the road, pedestrians walking away from the pedestrian crossing or changing direction abruptly).

Figure 5 shows how the model is capable of predicting pedestrian intention for different crossing scenarios in the intersection:

- C Pedestrian at the zebra crossing, ID: 42, 121, 162, 273;
- C Pedestrian at the bottom arm of the intersection, ID: 4, 100, 242;
- C Pedestrians on less utilized (i.e., walked) areas (left and top arms of the intersection), ID: 1, 171, 299.

Conversely, Figure 6 shows the classification results for non-crossing trajectories:

- Pedestrians walking on the sidewalk with *NC* intention, ID: 193, 369;
- *NC* while walking in front of the zebra-crossing, ID: 63, 77, 148, 183, 203_1, 242;
- Pedestrian walking away from the crosswalk, ID: 248;
- Pedestrian turning and *NC* in front of the road, ID: 203_2.

**Figure 7:**
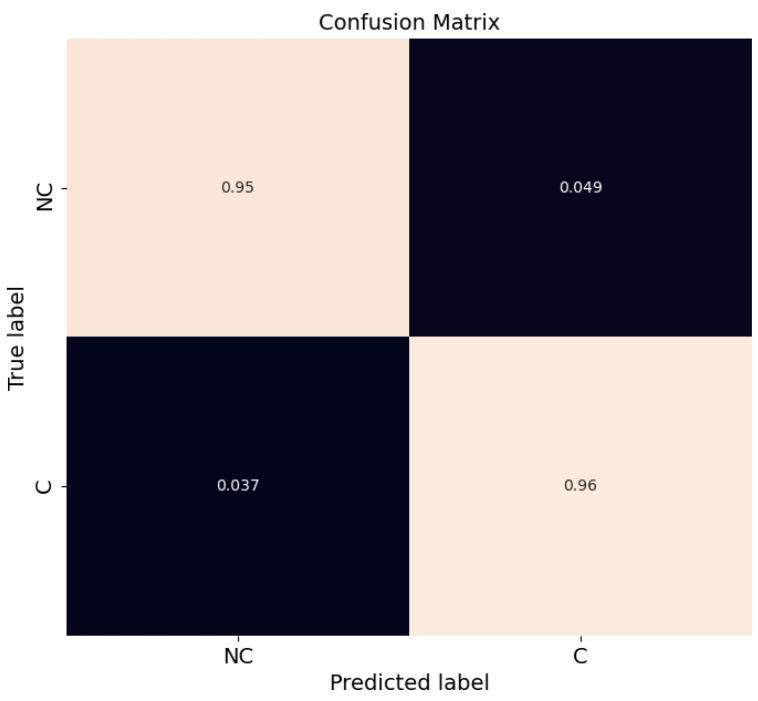
Confusion matrix for RF classification for all samples at recording #18.

Finally, for the sake of completeness, a confusion matrix of all trajectory samples for intersection recording number 18 is provided in Figure 7. Results show that the model is able to classify *C* and *NC* trajectory samples successfully, with TPs and TNs counts of 96% and 95%, respectively. Although there has been an increase in the number of FNs and FPs with respect to Figure 4, their values are below 5%.

To further evaluate the proposed model, we compare the classification accuracy of the RF against a NN. We train a multi-layer perceptron (MLP) [67] which stands for a NN with one or more hidden layers [68], by using the same training and testing sets discussed in section IV (B). In order to tune the hyperparameters of the NN, we use grid search (GS) [69] which let us define a search space grid of hyperparameter values and evaluate every position in the grid to find the best combination that achieves the higher classification accuracy. In our case the (GS) is parameterized for training a MLP classifier using 5-fold cross-validation with the following hyperparameter values:

- *Number of hidden layers* (1, 2, 3)
- *Number of units per layer* (10, 100, 200, 500, 1000)
- *Activation function* (rectified linear unit (ReLU), hyperbolic tangent)
- *Alpha* (0.0001, 0.05) [70]
- *Learning rate* (constant, adaptive) [70]
- *Solver* (stochastic gradient descent, Adam [71])

The best combination of hyperparameters is shown in Table 1 and achieves a mean accuracy of 0.97, which is moderately lower than the accuracy obtained by the RF. Both models are implemented in Python running on an Intel Desktop (Intel Core i7-8700 CPU @3.20 Ghz, 16 GB RAM). The training time of the RF is 6.9 s, which is significantly lower than the 861.2 s required to train the NN. Finally, the RF did not require a grid search, which took 28,150 s (~7.8 h) for a NN on the desktop system.

### 4.4. When Pedestrian Intention Is Not Clear

**Figure 8:**
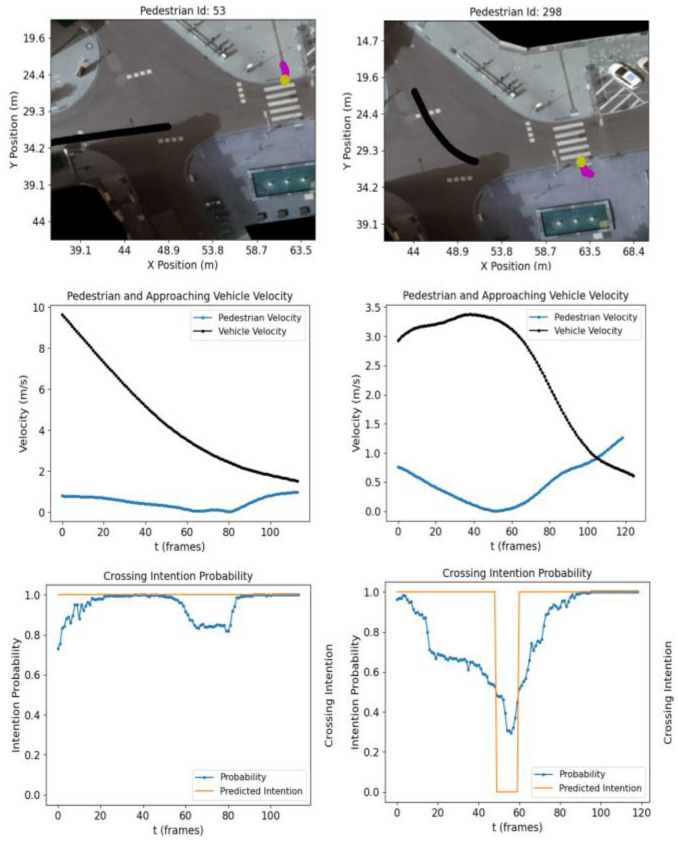
Intent prediction and velocity (plus approaching vehicle velocity) for pedestrians 53 (**left**) and 298 (**right**) from recording #18.

The previous section investigated the crossing intention in cases where the pedestrian maintained a steady walking velocity. This section studies how the model reacts when the pedestrian demonstrates apparent hesitation (e.g., a significant velocity drop before crossing). Figure 8 shows the crossing intention of two pedestrians that show hesitation before crossing the street (i.e., C intention). In order to investigate the potential impact of oncoming traffic, an extra plot displaying both the pedestrian (blue) and approaching vehicle (black) velocities is added.

Analysing the results in Figure 8 shows that the model reacts with a decrease in crossing intention probability for pedestrians ID 53 and 298 when the pedestrian velocity drops below 0.1 m/s. In the case of pedestrian ID 298, the probability goes below the decision boundary predicting *NC* for ~12 frames. Other features such as heading, position and distance to the road are used to infer pedestrian crossing intention. Pedestrian ID 298 changes direction and speed suddenly and therefore distance to the road, which contributes to the probability decrease seen in the initial frames (frames 1–20). A similar sudden change of direction with the consequent probability decrease is also observed for pedestrian ID 369 in Figure 6.

Finally, in an attempt to infer the cause of the significant pedestrian change of velocity before crossing, we further inspect the scenarios for pedestrians ID 53 and 298 by extracting the trajectory and velocity of the closest vehicle that is about to encounter the pedestrian. Pedestrian ID 53 decreases their velocity as the vehicle approaches the zebra crossing at above 9 m/s. The pedestrian velocity drop is lower at the beginning, possibly due to the distance of the vehicle to the zebra crossing and, although the driver is decreasing the vehicle speed, potentially encouraging the pedestrian to cross, we observe a pedestrian velocity drop (below 0.1 m/s) up to frame ~80 when the vehicle speed is below ~3 m/s. In contrast, the vehicle approaching pedestrian ID 298 does not decrease its velocity until frame ~40, forcing the pedestrian to decrease their velocity more aggressively. This supports the importance of studying pedestrian interaction with other agents in the scene. The model could also be improved by adding other contextual information (e.g., extracting further pedestrians features such as body pose or gaze direction).

## 5. Conclusions and Future Work

This paper has presented a random forest classifier to predict pedestrian crossing intention at a busy inner-city intersection. Using pedestrian features such as position, velocity and heading, provided by a publicly available dataset, and derived features, the model was able to predict crossing and non-crossing intention within a 3-s time window and an accuracy of 0.98. Due to the nature of the dataset used, providing only information related to the trajectory of the tracked pedestrian, detailed features such as body pose or gaze direction that might potentially improve the model performance could not be extracted. In future, this could be improved by building a dataset recorded from a fixed sensor and extracting these features. The authors also conducted a comparison analysis between an RF and a NN showing that the RF outperformed the latter in terms of accuracy and training times. Scenarios where the crossing intention was not clear were also investigated (e.g., pedestrian slowing down before crossing), revealing the potential importance of studying pedestrian interaction with other agents in the scene in future work.

## Figures and Tables

**Table 1:** Grid search results.

Hyperparameter	Value
Number of hidden layers	3
Number of units per layer	1000
Activation function	ReLU
Solver	Adam
Alpha	0.05
Learning rate	Constant
